# Road dust biases NDVI and alters edaphic properties in Alaskan arctic tundra

**DOI:** 10.1038/s41598-018-36804-3

**Published:** 2019-01-18

**Authors:** Daniel E. Ackerman, Jacques C. Finlay

**Affiliations:** Department of Ecology, Evolution, and Behavior, University of Minnesota, St. Paul, MN United States

## Abstract

Increased road-building activity in the arctic has the potential to impact adjacent ecosystems. Roads in permafrost regions are often built atop insulative gravel pads that generate dust plumes, altering soil chemistry and ecosystem function of nearby tundra. Here, we measure edaphic and vegetation characteristics along transects of decreasing dust deposition perpendicular to the Dalton Highway in northern Alaska. We quantify the impact of dust deposition on normalized difference vegetation index (NDVI), a proxy for aboveground plant biomass. Deposition of calcium carbonate-rich dust declined from 1.625 grams m^−2^ day^−1^ immediately adjacent to the road, to negligible levels 625 meters away. Along these transects from the road, we found declines in soil moisture and temperature, thaw depth, shrub height, and foliar nitrogen content, indicating that tundra roads create corridors with edaphic conditions favorable to vascular plant growth. At sites nearest the road, dust deposited on leaf surfaces reduced measured NDVI values by 0.24 by blocking reflectance properties of the underlying leaves. Our findings on the impacts of roads and dust deposition on adjacent tundra may aid planning of future infrastructure projects. We caution that dust deposition may negatively bias NDVI-based estimates of plant biomass, especially where unpaved roads are common.

## Introduction

Roadways alter ecological processes through acoustic disturbance, edge effects, and human-aided dispersal^1–5^. In permafrost regions, where roads are built on gravel pads to prevent thermal erosion of permafrost, road dust deposited in the tundra is among the most widespread direct human impacts on ecosystems^6–8^. As natural resource development, climate change, and tourism spur the proliferation of permanent roads in arctic tundra^9–11^, an enhanced understanding of road dust effects on biodiversity, structure, and function of native tundra ecosystems is urgently needed^12–16^.

Chemical properties of road dust resemble the source material from which the road gravel was quarried. Often, source material geochemistry contrasts sharply with the environment where road dust is deposited, with consequences for locally-adapted communities and ecosystems. For example, deposition of macronutrients such as nitrogen or phosphorus may stimulate primary productivity and favor plant species with relatively low nutrient use efficiencies including deciduous shrubs^17,18^. Further, deposition of material that generates dissolved carbonates in soil solution may alter pH, a vital determinant of plant species richness and community assembly in arctic tundra^19,20^.

Alaska’s Dalton Highway crosses moist acidic tundra (MAT) north of the Brooks Range, where road material is quarried from calcareous limestone bedrock^6,21^. The Dalton Highway was constructed in 1974 to support the Trans Alaska Pipeline System. A series of studies published between 1980 and 2006 details how sustained deposition of calcareous road dust alters edaphic properties and plant community composition in MAT adjacent to the Dalton Highway^12,22–24^. Walker and Everett^12^ found that low-albedo dust induced early spring snowmelt in tundra adjacent to roads, increased thaw depth, and eliminated acidophilic taxa including *Sphagnum* moss. Auerbach *et al*.^22^ expanded this analysis by demonstrating that dust effects on plant communities are stronger in MAT than in moist non-acidic tundra, because road dust increased MAT soil pH from 4.0 to 7.3 and reduced roadside species richness by 50%. Resampling the same plots as Auerbach *et al*.^22^. Myers-Smith *et al*.^23^ showed that similar community impacts persisted a decade later and that soil pH continued to rise. Since then, there has been little study of road dust impacts on soil characteristics in this region. This lack of information is significant, given that edaphic properties (e.g. soil moisture and temperature, thaw depth) affect both plant communities and the stability of Dalton Highway infrastructure itself.

In addition to potential edaphic effects, road dust deposition alters tundra reflective properties used to characterize plant community structure, function, and physiology^25,26^. These remote sensing impacts have received sparse attention compared to more commonly analyzed responses to road dust, such as edaphic changes and plant community shifts^12,22,23,27,28^. Analyses of multispectral SPOT imagery and airborne radiometry demonstrate that the spatial extent of dust deposition on tundra can be estimated remotely through changes in certain spectral bands, including those used to calculate ecologically useful indices such as NDVI^29,30^. Such spectral patterns could arise in two different ways. First, dust deposited on the leaf surface could alter plant physiology, e.g. by moderating light intensity, clogging stomatal pores, or changing leaf thermal regime. Alternatively, the spectral properties of the dust itself could mask those of the underlying leaf, causing spectral measurements to reflect properties of the dust rather than the targeted leaf. This potential masking effect of road dust may lead to underestimates of productivity in tundra, since remotely sensed NDVI is commonly used to estimate plant productivity in arctic tundra^31–34^.

In this study, our first aim is to expand the descriptive record of roadway effects on tundra adjacent to the Dalton Highway, emphasizing edaphic variables relevant to both arctic ecosystems and highway infrastructure. Our second aim is to test the “masking” hypothesis, i.e. that dust deposition onto canopy leaves alters NDVI because dust blocks the sensing of the true spectral properties of the underlying leaf. This hypothesis would be supported if both following criteria were met:

1. Dust deposition rates correlate with leaf-level NDVI.
2. Dust removal from leaves, regardless of initial deposition rate, instantly (i.e. faster than leaf can physiologically adjust) brings NDVI up to baseline levels for the study region.

## Methods

### Aim 1: Description of roadway effects on tundra

During mid-July 2015, we sampled road dust deposition rate and chemical composition along three transects perpendicular to the Dalton Highway to the east, matching the prevailing wind direction. Mean summer wind speed in the region for the summer of 2015 was 2.80 m/s (standard error 0.03; supplementary Figure S1). We collected dust in open-topped plastic cylinders (6.35 cm diameter, 15 cm deep) affixed to rebar 50 cm above ground surface. The dust collectors were placed at five points along each transect, at distances from the road of 1, 5, 25, 125, and 625 m, for 48 hours during which no precipitation occurred. These distances were chosen to match previous models of exponential decline of dust deposition as distance from road increases^12,24^.

Following the dust collection period, we poured 100 mL of deionized water into each dust collector and placed it on a stir plate for 30 minutes to suspend all solids and dissolve ions. We then filtered each suspension through pre-weighed 0.7 μm glass microfiber filters. The filters were dried and weighed to find the total mass of dust deposited in each collector. Dried filters were also used to measure particulate phosphorus levels with molybdate colorimetry on a Varian Cary 50 Bio UV – Visible Spectrophotometer, following methods from Janke *et al*.^35^. Filtrate water from each sample was then analyzed for dissolved inorganic carbon (DIC, composed primarily of bicarbonate) and dissolved calcium respectively using a Shimadzu TOC-L CSN analyzer and a Thermo Scientific iCAP 6500 dual view Inductively Coupled Plasma – Optical Emission Spectrometer. Neither dissolved phosphorus nor nitrogen were measured for this study. Due to the 30-minute mixing period, measurements of the two dissolved constituents, DIC and calcium, represent short-term bioavailable pools of these elements. In contrast, the measurement of particulate phosphorus on the filters represents the pool phosphorus that may become bioavailable in soil solution over longer timespans via weathering. Simple linear regression was used to characterize the change in elemental pools along the transects.

To determine whether prevailing wind direction affects deposition rate, a similar collection was made at distances of 1, 25, and 125 m from the road on both the east and west sides of the highway in mid-July 2016, during a period when wind blew primarily, though weakly on average, to the northwest (supplementary Figure S1). We ran a multiple linear regression model with dust mass as the response variable, and distance from road and direction as predictor variables.

In addition to deposition rate, edaphic variables including soil moisture, soil temperature, and thaw depth were measured on both sides of the road in mid-July 2016, along with the height of the locally-dominant canopy species, *Salix pulchra*. Soil moisture was measured as percent volumetric water content at 20 cm depth. Temperature (°C) was measured at 12 cm depth. Thaw depth was measured with a drain tile probe. All edaphic variables were characterized as the mean (and standard error) of 25–30 replicate measurements of each variable at each distance reported.

Along each of the three transects to the west of the road in mid-July 2016, samples of *S. pulchra* green leaf tissue were collected from four individual plants adjacent to the road and at a reference site 625 m from the road. Leaf samples were dried and foliar nitrogen and carbon content were measured with a Costech ECS 4010 Elemental Analyzer. Foliar chemistry values from the two sites were compared with unpaired Student’s t-tests.

### Aim 2: Masking hypothesis

Leaf-level NDVI measurements were conducted in the lab on *S. pulchra* specimens collected along the three transects in mid-July 2015 during peak greenness. At each transect point, we collected the two closest *S. pulchra* specimens, cut them at the root collar, and kept the stems in water during transport back to the lab. Specimens were carried in slotted trays spaced to ensure adjacent plants did not contact each other and potentially rub dust off leaf surfaces. One leaf from the canopy of each plant was randomly selected for hyperspectral measurements (wavelengths between 310 nm and 1130 nm, in increments of 1 nm) with a Unispec (PP Systems, Haverhill, MA, USA) taken within 90 minutes of collection. Scans were made with the Unispec bifurcated fiber optic cable as the light source, which was fixed at a 60° angle to the leaf using the Unispec leaf clip. Integration time was calculated with Unispec’s “optimize integration” time algorithm. White reference measurements were made with a 99% reflectance standard (Spectralon, LabSphere, North Sutton, NH, USA). To quantify the effect of settled dust on NDVI, we took triplicate hyperspectral measurements both before and immediately after dust was removed from the leaf surface with a Kimwipe. Replicate measurements were averaged before calculating NDVI following the standard formula, described by Boelman *et al*.^36^. Our study design did not allow us to determine if (and how much) dust was removed from the leaf surface by wind prior to our collections.

## Results

### Description of roadway effects on tundra

**Figure 1:**
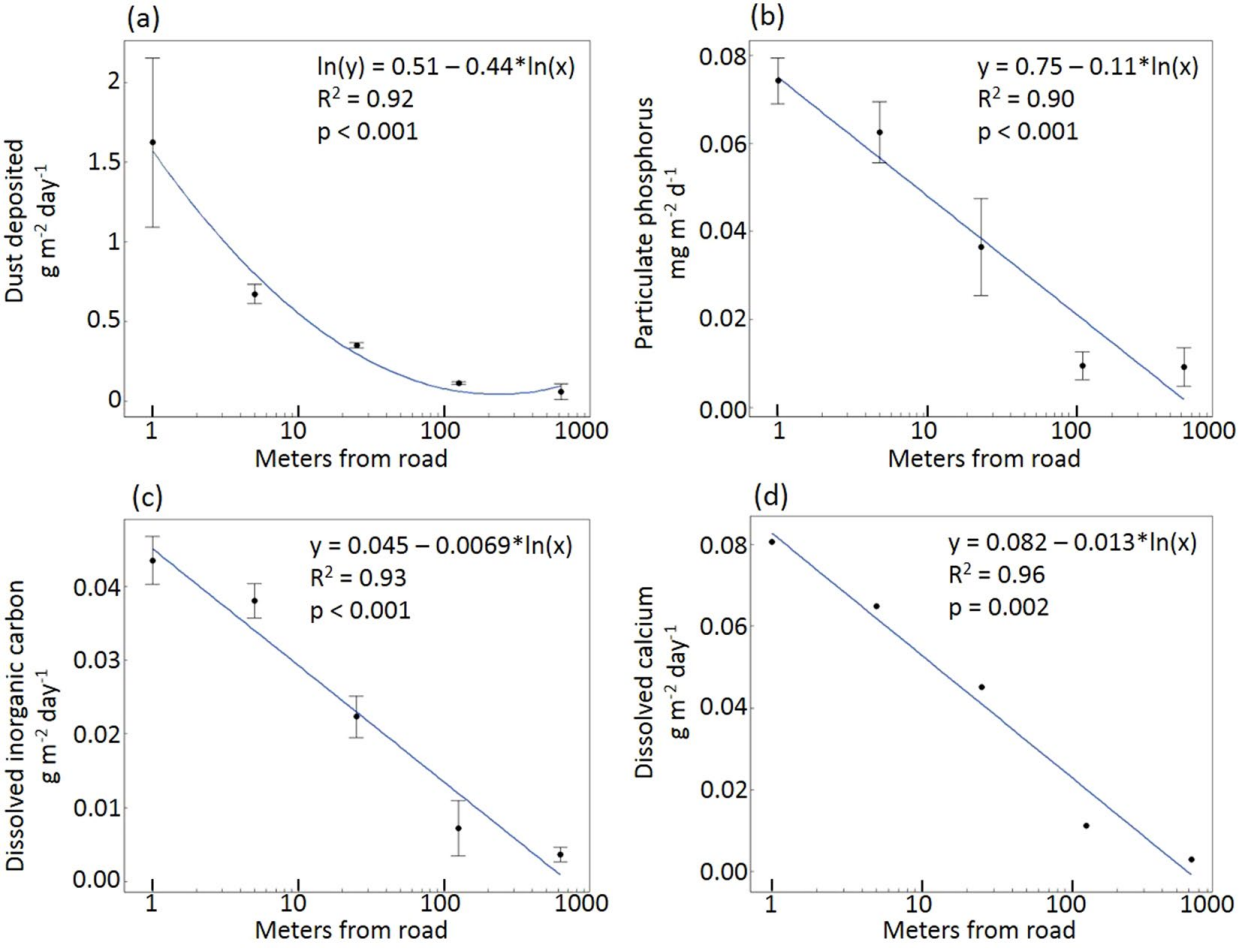
Dust deposition and its constituent chemical components decline with increasing distance from road. Panels show total mass (**a**), mass of particulate phosphorus (**b**), mass of immediately available dissolved inorganic carbon after 30 minutes of mixing with deionized water (**c**), and mass of immediately available dissolved calcium after 30 minutes of mixing with deionized water (**d**) from dust collected at sites from 1 m to 625 m to the east of the road during a 48-hour period in July 2015. Each panel contains simple linear regression equations detailing the relationship between that panel’s y-axis variable and distance from road (x).

Dust deposition rates measured in the 2015 survey matched predictions of exponential decline with distance from road. The greatest deposition rates were found at sites 1 meter from the road, with dust loads of 1.625 grams m^−2^ day^−1^ (standard error = 0.375). Dust deposition at the remote sites 625 meters from the road was just 0.061 grams m^−2^ day^−1^ (standard error = 0.030). Deposition of individual chemical constituents, including particulate phosphorus, dissolved calcium, and inorganic carbon, matched the spatial patterns found in the dust deposition measurements (Fig. 1).

**Figure 2:**
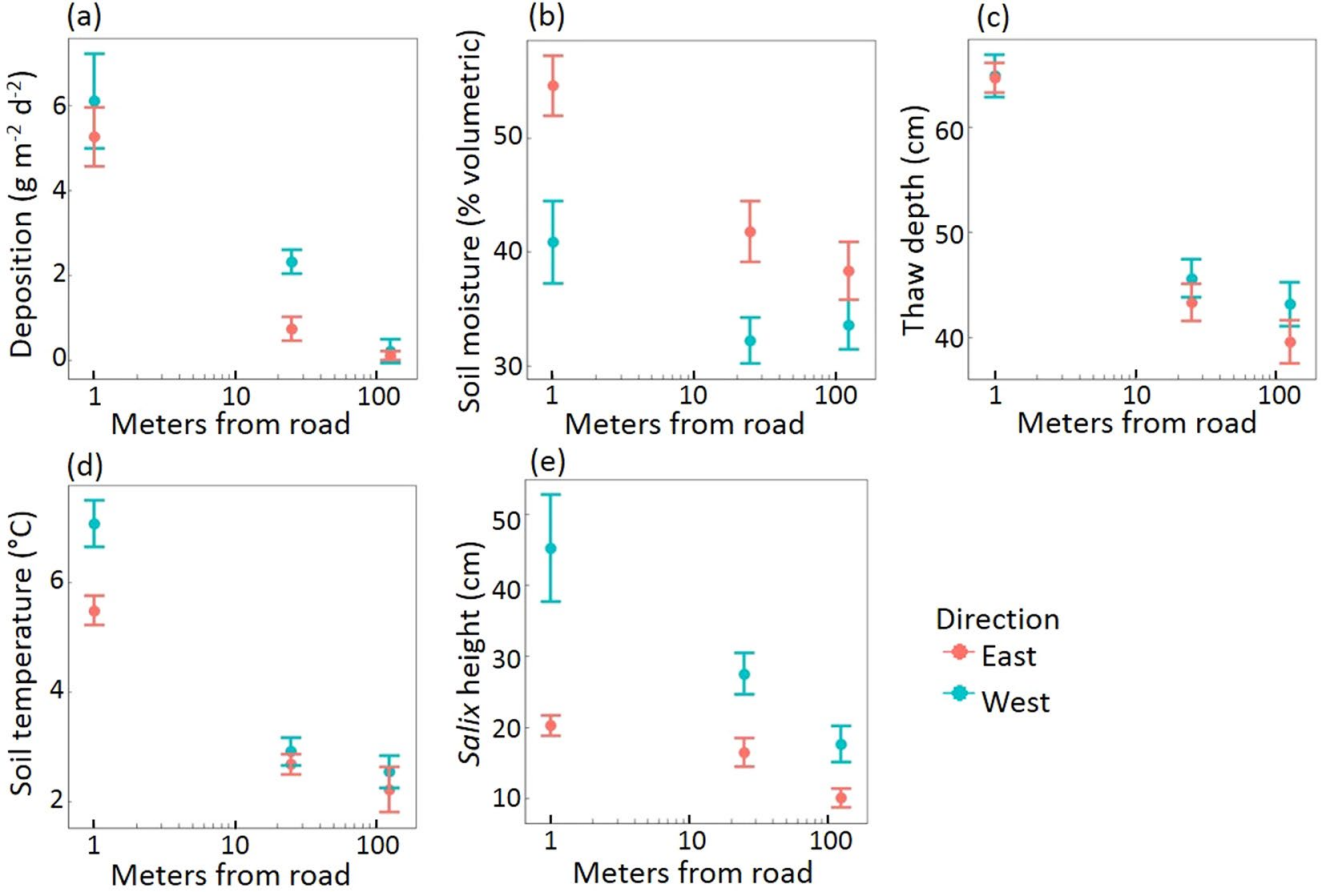
Edaphic variables and shrub height decline with increasing distance from road. Panels display dust deposition rate (**a**), soil moisture (**b**), thaw depth (**c**), soil temperature (**d**), and *Salix pulchra* shrub height (**e**) for sites at 1 m, 25 m, and 125 m from the road to the east (red) and west (blue) in July 2016. Soil moisture was measured at 20 cm depth and soil temperature was measured at 12 cm depth.

Edaphic characteristics measured in the 2016 survey indicated that proximity to road provided favorable conditions for the growth of shrubs (Fig. 2). Soils near the road were warmer, more moist, and had a deeper active layer. Mean *Salix pulchra* height and stem diameter were also greater near the road. Dust deposition rate did not depend on direction from road (p = 0.31). *S. pulchra* adjacent to the road had greater foliar nitrogen content and a lower carbon-to-nitrogen ratio than *S. pulchra* further from the road (Table 1).

**Table 1:** Foliar nitrogen and carbon concentrations and carbon-to-nitrogen rations in green leaf tissues of *Salix pulchra*.

	1 m from road	Reference site	p-value
% Nitrogen	2.35 (0.20)	1.65 (0.09)	0.027
% Carbon	50.48 (1.07)	48.00 (1.30)	0.194
Carbon:nitrogen	21.86 (2.31)	29.36 (1.09)	0.031

Sites adjacent (1 m) to the road are compared with reference sites (625 m) far from the road. Mean (standard error) values are shown, along with the p-value associated with an unpaired Student’s t-test.

### Masking hypothesis

**Figure 3:**
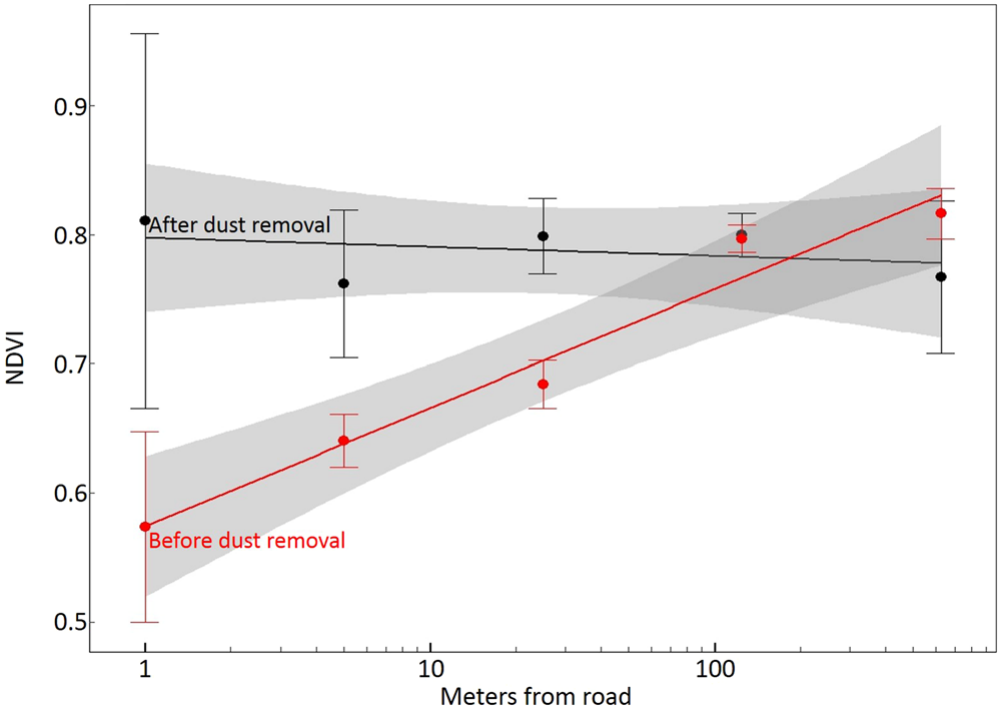
Dust removal from leaf surface increases NDVI. Paired leaf-level NDVI measurements are shown before (red) and after (black) dust removal at distances from 1 m to 625 m away from the road. Post-removal measurements were constant across the transects, while pre-removal measurements increased with distance from the road. The lines show slope estimates with standard error (gray bands) for a simple linear regression of each set of NDVI measurements on distance from road. The impact of dust removal on NDVI was not detectable for the two sites furthest from the road (125 m and 625 m).

**Figure 4:**
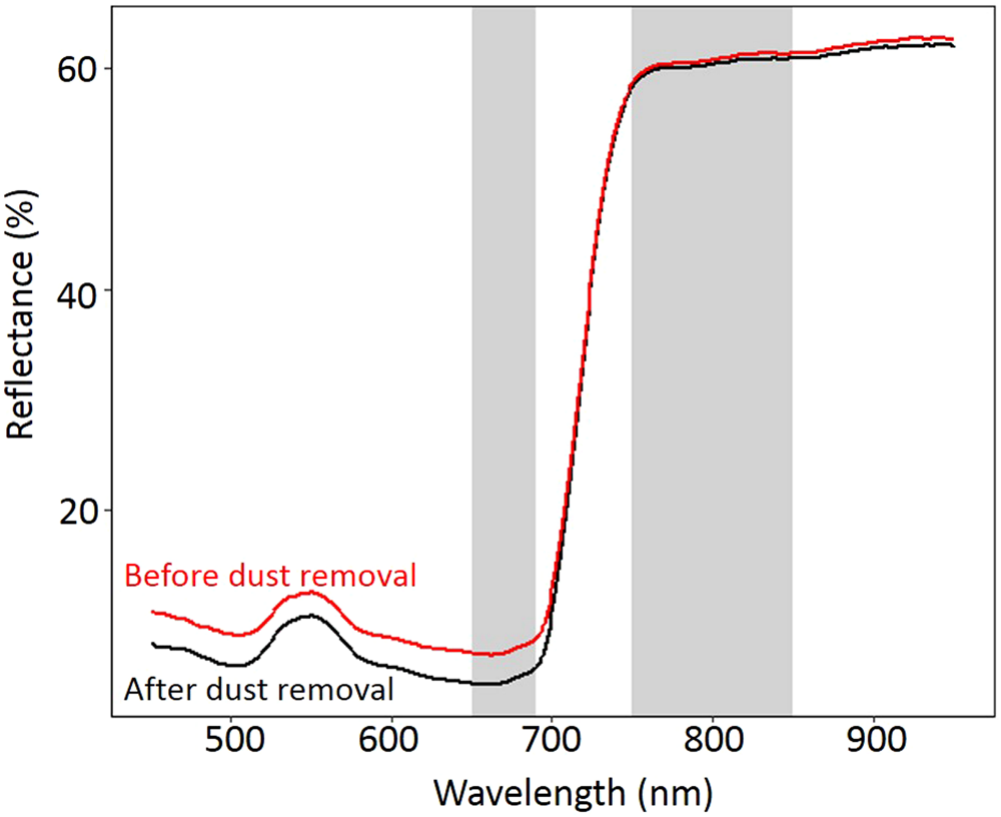
Dust removal from leaf surface decreases reflectance of photosynthetically active radiation (400–700 nm). Reflectance spectra are shown for a *Salix pulchra* leaf sampled 1 m away from the road before (red) and after (black) dust removal. The two spectral measurements were taken 5 seconds apart. Gray bands highlight the wavelengths used to calculate NDVI.

Dust deposition load showed a significant negative exponential relationship with NDVI, explaining 98% (p < 0.001) of variation in leaf-level NDVI. Dust removal from all leaves, regardless of distance from road, instantly shifted NDVI to values similar to reference sites not impacted by dust deposition (Fig. 3). At sites 1 meter from the road, where dust deposition rate was greatest, dust removal from the leaf surface increased NDVI by 0.237 (standard error = 0.069), while dust removal had no impact on NDVI at reference sites 625 meters from the road. For leaves sampled from high deposition sites, dust removal reduced reflectance throughout the 400–700 nm wavelength range (Fig. 4).

## Discussion

### Description of roadway effects on tundra

We found that dust deposition declined exponentially with distance from road, in a pattern and magnitude consistent with prior research^12,24^. This decline in total mass deposited matched the patterns of several chemical components of dust deposited along the transects in 2015, particularly phosphorus and calcium, elements with the potential to impact plant productivity and communities. While nitrogen is the principal limiting nutrient to terrestrial primary productivity in moist tundra, phosphorus is often colimiting in moist tundra and may be limiting in wet sedge tundra^21,37,38^. Further, there is longstanding evidence that calcium deposition levels similar to those documented here can alter both individual plant growth and community composition, especially in acidic soils^39–42^. These levels of calcium, and bicarbonate, are also the probable drivers of the observed pH increase and *Sphagnum* elimination in roadside tundra over the last 40 years^12,22,23^. Though we did not measure soil nutrient concentrations for this study, we did find that *S. pulchra* foliage directly adjacent to the road had greater nitrogen content and lower carbon-to-nitrogen ratios than foliage at reference sites 625 meters away (Table 1). Due to the greater thaw depth near the road, roadside vegetation may have access to greater soil volume for nutrient acquisition. Soil nitrogen availability may also be greater on a per-volume basis closer to the road thanks to warmer ground temperatures (Fig. 2), which stimulate mineralization. Although we were not able to measure all changes in nutrient availability associated with dust deposition, foliar nitrogen, dust phosphorus data, and warmer soil temperatures together indicate that nutrient availability was substantially higher near the roadway.

Though a full consideration of road dust effects on aquatic systems is beyond the scope of this study, road dust may partially contribute to the observed alkalinity increase over the last 40 years in surface waters near the Dalton Highway, in addition to factors such as subsurface permafrost thaw^43–45^. Our analysis revealed high loads of bioavailable calcium and bicarbonate (a major component of alkalinity) in the dust, whose delivery to surface waters may be augmented with runoff from roadways. Further, the limestone bedrock from which road material is quarried has a low strontium stable isotope ratio (^87^Sr/^86^Sr), which is consistent with the reduced ^87^Sr/^86^Sr ratio measured in local surface waters since 1994^43,44,46,47^. Calcareous road dust contribution to increased alkalinity in tundra surface waters has previously been documented along the Dempster Highway in northern Canada^48^.

We found that proximity to road yielded edaphic conditions more favorable for vascular plant growth; soils near the road were warmer, moister, and had a deeper active layer. The patterns in soil temperature and active layer depth are consistent with prior studies along the Dalton Highway^12,23^, which found that growing season soil temperatures were elevated adjacent to the road where *Sphagnum spp*. was eliminated by dust-induced decreases in acidity. However, soil moisture patterns in our study contrast with those of Auerbach *et al*., who found drier soils near the road^22,49^. While we cannot pinpoint the source of these discrepancies, our finding of elevated roadside soil moisture may be due to the impoundment of water at the base of the roadbed near our sampling sites. Alternatively, changes in roadside soil moisture through time may relate progressive thaw and slump dynamics caused by road impacts on local thermal regimes. Coupled thermal and morphological dynamics have been demonstrated near roads at the Prudhoe Bay Oilfields and the Dempster highway^15,49^. Energy balance effects of the low-albedo road surface may also contribute to elevated roadside soil temperatures.

Observed edaphic patterns of increased temperature, thaw depth, and resource availability near the road positively affected size of a dominant shrub species. *S. pulchra* were tallest adjacent to the road, and shrub height declined with distance from road. This finding aligns with results from Cameron and Lantz^50^, who found that roadside proliferation of tall shrubs near the Dempster Highway was linked to increased soil moisture. In our study region, Myers-Smith *et al*.^23^ found no correlation between areal deciduous shrub cover and proximity to road, but they did not measure size of individuals. Increased shrub height along riparian corridors is believed to have facilitated recent range expansion of moose and snowshoe hare beyond the boreal forest into the tundra biome^51,52^. Tall shrub establishment along roadside corridors could have similar consequences for wildlife habitat. Sustained increases in shrub size adjacent to the Dalton Highway may also impact ecosystem function and aesthetic value^28,53^, effects that should be considered in environmental impact assessments of future road proposals in tundra regions.

### Masking hypothesis

Our NDVI results appear to support the masking hypothesis, as both key criteria were met. First, dust deposition load correlated strongly with leaf-level NDVI. Second, dust removal from the leaf surface, regardless of initial deposition load, immediately brought NDVI up to reference levels for the region. Therefore, it is likely that road dust deposited on leaves lowers NDVI at this site by blocking spectral properties of the underlying leaf.

In addition to spectral masking, dust could impact leaf physiology. For example, dust particles could impede gas exchange via stomatal clogging^54,55^. This mechanism was likely not a significant factor in our study, because dust was removed just from the top of the leaf, not the bottom where most stomata are located. Dust could also reduce incident light on the leaf’s surface or alter thermal regimes^55,56^. Direct tests of these potential mechanisms were beyond the scope of our study but could be important in determining overall ecosystem effects of dust deposition in tundra.

Since dust deposition was shown to reduce leaf-level NDVI, we suggest that the masking effect of dust deposition may negatively bias NDVI-based estimates of plant biomass and productivity. Dust appears to mask greenness in the leaf-level NDVI signal, and likely affects other spectral indices as well. This bias may be particularly problematic in the arctic, where gravel roads are common and spectral indices are often used as proxies for productivity^32–35^, but has relevance wherever anthropogenic dust impacts ecosystems. For example, experimental ecological field sites are often accessible by gravel roads, where certain plots may be more influenced unintentionally by dust deposition than others. Further, high-value agricultural regions are often located in areas with high concentrations of dirt roads, where crop monitoring programs may be influenced by dust deposition.

The leaf-level NDVI pattern we found (Fig. 3, red line) does not imply that the same pattern holds at larger spatial scales. For example, airborne measurements may show that canopy-level NDVI *declines* with distance from road, corresponding with declines in standing biomass and leaf-area index. However, we argue that dust masking could alter the *slope* of such a decline by negatively biasing NDVI values near the road where dust is most prevalent. The strength and spatial scale of such a bias remains to be tested at the canopy level.

### Future directions

We found that conditions near the Dalton Highway in Northern Alaska were favorable to vascular plant growth, as roadside soils were warmer, moister, had a deeper active layer, and received greater deposition of nutrients than soils at remote reference sites. Consequently, shrub height and foliar nitrogen content declined with distance from the road. Further, our results supported the “masking hypothesis,” which states that dust deposition onto leaves reduces NDVI by blocking spectral characteristics of the underlying leaf. The masking effect of dust appears to be strongest for the range of photosynthetically active radiation, between 400 and 700 nm. We call for the continued monitoring of dust effects on tundra ecosystems, especially across multiple plant communities types where dust-induced chemical changes may have variable effects. Such knowledge is necessary for predicting ecological effects of a likely future increase in the construction of high-latitude infrastructure^11^. Finally, we caution that road dust deposition may negatively bias NDVI-based estimates of vegetation biomass, particularly in permafrost regions and rural areas where dirt roads are common. Future study relating leaf-level observations to plot- and landscape-level NDVI patterns are needed. Achieving these future research goals will require a combination of ground-based sampling and remote sensing measurements.

## Electronic supplementary material

Supplementary Figure S1

Dataset 1

## Data Availability

Data generated through this project are available in the supplementary information accompanying this publication.
