# Supplementary material for: Road dust biases NDVI and alters edaphic properties in Alaskan arctic tundra
Source: Sci Rep. 2019 Jan 18;9:214. doi: 10.1038/s41598-018-36804-3 (PMC6338752; doi:10.1038/s41598-018-36804-3)
Supplement: Supplementary file 1 — Supplementary Figure S1 [file 41598_2018_36804_MOESM1_ESM.pdf]

Supplementary figure S1 for: Road dust biases NDVI and alters edaphic properties in Alaskan arctic tundra

Authors: Daniel E. Ackerman and Jacques C. Finlay

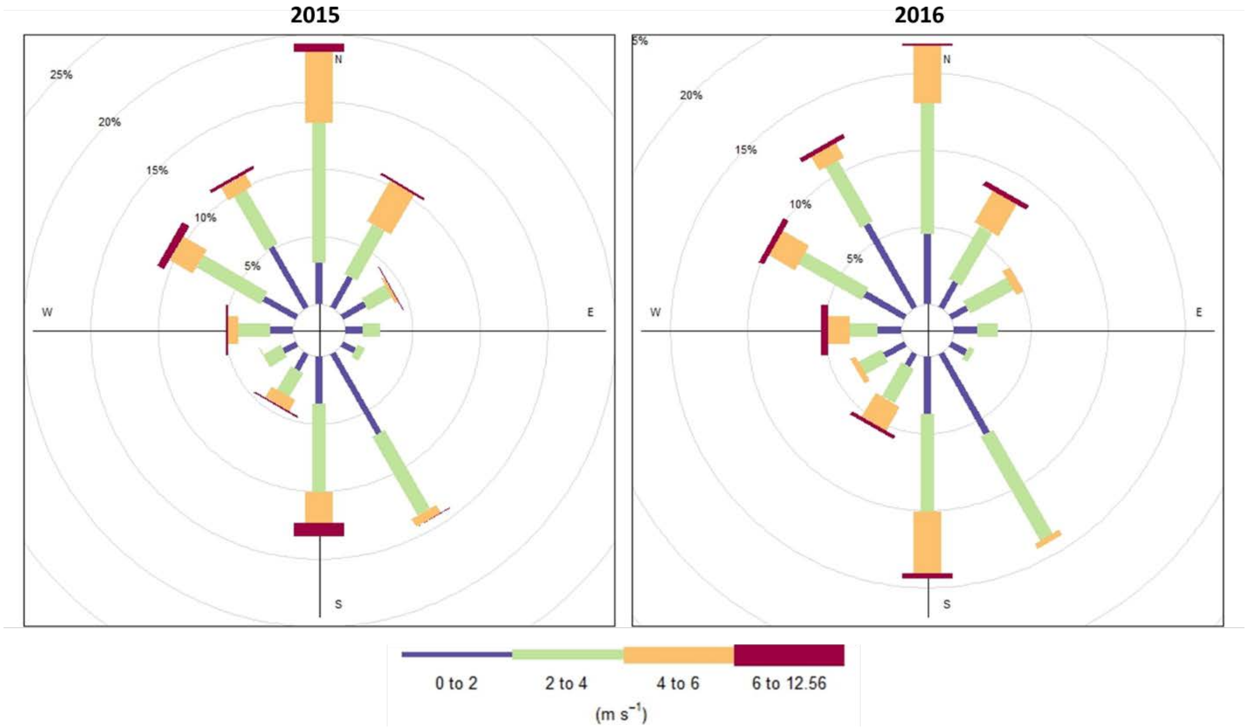

Supplementary figure S1. Wind roses for the summers of 2015 (left) and 2016 (right) at Toolik Field Station (68° 38’ N, 149° 36’ W). Data include hourly wind speed and direction readings at 5 m between June 1 and August 31 each year, provided by the Toolik Field Station Environmental Data Center. Concentric circles indicate the percentage of total readings that fall into each of the 12 directional bins displayed. Figures were created using the R package ‘openair’ version 2.5-0 by Carslaw and Ropkins (2018; https://cran.r-project.org/web/packages/openair/index.html).
